# Multiband antenna design with enhanced radiations using characteristic mode analysis

**DOI:** 10.1038/s41598-023-44923-9

**Published:** 2023-10-19

**Authors:** Dilaawaiz Fazal, Qasim Umar Khan, Ic-Pyo Hong

**Affiliations:** 1 Smart Natural Space Research Center, Kongju National University, Cheonan, 31080 South Korea; 2 Department of Electrical Engineering, National University of Sciences and Technology, Islamabad, 44000 Pakistan; 3 Department of Smart Information Technology Engineering, Kongju National University, Cheonan, 31080 South Korea

**Keywords:** Engineering, Electrical and electronic engineering

## Abstract

In this research, a multiband patch antenna design based on the superposition of multiple modes at the same resonant frequency is presented. The concept of the contribution of lower order modes (LOMs) with the higher order dominant modes (HODMs) is investigated using characteristic mode analysis (CMA). In order to provide similar broadside radiations in three bands, the radiating capability of the LOMs is enhanced in the resonant frequencies of HODMs. These HODMs when excited alone provide null in the broadside radiations of the antenna. Using a single feed, enhancement in the broadside radiations of the antenna is achieved with the superposition of multiple modes at the same resonant frequency. Based on the proposed concept two antennas have been designed and fabricated. The antenna provides stable and enhanced radiations. simulated and measured results are in good agreement. These antennas find application in many applications including communication systems, base station antennas, 5G communications, satellite communication etc.

## Introduction

With the advent of modern wireless communication^1, 2^ miniaturization of devices increased the demand for a single antenna system working in multiple bands^3–5^. Modern communication devices need an antenna to be low-cost, multiband, and smaller in size. Various techniques to achieve multiband antenna operation have been reported in the literature, where additional modes within a band of interest are introduced by using: fractals^3–10^, split ring resonators^5^, U-slots, vias and varactor diodes^11–14^, use of dual-feed and more than one radiating element^14^, slot meandered patch^15^ and V-shaped antenna configuration^16^, mobile handset antennas^17, 18^, metamaterial loaded antenna^19^, and slotted ground^20^. Patch antenna provides attributes of low cost, compactness, and conformability, making it a suitable candidate for modern antenna systems. However, the patch antenna suffers from the nulls in the radiations and grating lobes due to the undesired radiations offered by the higher order modes^21–24^, consequently making it very difficult to achieve desirable radiations in multiple bands of an antenna. Multiband and dual-band antennas with nulls in the radiations cannot be used in applications where broadside radiations are required in all bands^25, 26^. To achieve broadside radiations in the second band, slots are incorporated along the radiating edges of the rectangular patch to perturb $TM_{30}$ mode^21^. A slight dip in the radiations of $TM_{20}$ mode of the triangular patch antenna^24^, is removed by introducing slots in^22^ which provided current perturbations, thereby making perturbed $TM_{20}$ modal currents similar to the modal currents of $TM_{10}$ mode. Metamaterial loading is also used to remove null in the $TM_{20}$ mode of a rectangular patch antenna, hence making it usable for the dual-band antenna operation which requires broadside maximum radiations at both resonant frequencies^27^. Higher order $TM_{21}$ and $TM_{31}$ modes are excited in circular patch antenna by the slot-loading technique^28^. Patch and monopole modes in a shared aperture are also used to excite two modes for dual-band operation^29^.

Lately, the theory of characteristic modes (TCM) gained popularity in solving antenna design problems. TCM provides complete information about radiating modes of an arbitrarily shaped PEC structure in the range of frequencies under consideration^30–32^. This attribute makes it an excellent antenna analysis tool to identify radiating modes inherent to the structure. Various efforts have been made by the researchers to design dual-band and triple-band antennas using characteristic mode analysis (CMA), including multiband MIMO antennas^33–36^, fractal shaped dual-band antenna^37^, U-slot loaded triple-band antenna^13^, loading triangular slot^38^, vias^13, 39^ and metasurfaces^39^ to introduce additional modes within a band of interest.

For the multiband antenna operation using CMA, multiple radiating characteristic modes (CMs) are required to be excited in the band of interest. The number of CMs is directly proportional to the size of a radiator; hence density of modes increases with the increase in frequency. The radiation patterns of an antenna are shaped by the radiations of DMs, consequently, for a multiband antenna with usable broadside radiations, dominant modes (DMs) within the band should provide usable radiations. Furthermore, coupling of undesired modes with the source also deteriorates the radiation performance of the antenna in higher bands. Some of the latest reported research work by Chen et al.^40, 41^ is based upon the removal of higher order modes (HOMs) using CMA to enhance antenna radiations. In^40^ an unwanted HOM is suppressed to enhance the realized gain of the antenna by using a metasurface in front of the antenna array, in^41^ suppression of HOMs is carried out by inserting vias and slots in the unit cells of a metasurface structure to improve performance of the antenna in terms of radiations. Nevertheless, very few publications are related to the pattern enhancement by manipulation of HOMs in CMA. To the author’s knowledge, none of the published work is related to the suppression or contribution of LOMs along with DMs to enhance antenna radiations. This work presents the intentional excitation and superposition of multiple LOMs with the HODMs to remove radiation nulls in the higher bands. The significance of the LOMs is enhanced beyond their resonant frequencies, and the superposition of non-significant LOMs with the HODMs at the same frequency is carried out to enhance antenna radiations. It is found that the excitation of multiple modes at the same resonance provides more control over shaping the antenna’s radiation characteristics. The proposed technique is applied to the Partial Koch fractal structure reported in^42^, which suffers from the null in the radiations in the second band. Current authors introduced pi-shaped slots on the surface of the structure as reported in^43^, however, perturbations produced by the slots only removed radiations nulls in the H-plane of the second band. To show the problem of radiation nulls, and provide the superiority of the proposed technique, a multiband antenna using the excitation of DMs in their respective bands is also presented.

## Theory of characteristic modes

TCM provides complete information about the resonant behavior and radiating mechanism of an arbitrary shaped conducting structure. Deep physical insight into the radiating mechanism is provided in terms of modal currents and parameters such as eigenvalue $\lambda _{n}$, characteristic angle $\alpha _{n}$, and modal significance (MS) values. The magnitude of the eigenvalues provides information about the radiating capability of a mode. The closer the eigenvalue of the respective mode to zero the better it radiates^30–32^. Eigenvalue is given by:

1 $$\begin{aligned} \lambda _{n} = \dfrac{P_{reac,n}}{P_{rad,n}} \end{aligned}$$

Modes with $\lambda _{n}=0$ are resonant modes, $\lambda _{n}>0$ are inductive modes, whereas modes with $\lambda _{n}<0$ are capacitive modes. eigenvalues range from $\left[ +\infty ,-\infty \right]$.Due to the infinite range of eigenvalues, the other two parameters are preferred to identify the radiating modes of a structure. MS and $\alpha _{n}$ are better alternatives hence the representation of eigenvalues in terms of characteristic angles is derived in^30^, as given by

2 $$\begin{aligned} \alpha _{n} = 180^{\circ } -tan^{-1}\lambda _{n} \end{aligned}$$

$\alpha _{n}$ varies in the range of $\left[ 90^{0},270^{0}\right]$. Modes with $90^{0}<\alpha ^{n}<180^{0}$ are inductive, modes with $180^{0}<\alpha ^{n}<270^{0}$ are capacitive, whereas modes with $\alpha _{n}=180^{0}$ are good radiators.

MS^31^, represents the contribution of a mode to the total antenna radiations when an external source is applied, given by:

3 $$\begin{aligned} MS = \bigg | \dfrac{1}{1+j\lambda _{n}} \bigg | \end{aligned}$$

Equations (2), (3) as being derived using eigenvalues, provide a better representation of CMs. Due to the small range of values $[0-1]$, MS is considered to be the most convenient way to identify radiating modes. The closer the MS value of a mode to 1, the better it radiates. Modes having $MS\ge \dfrac{1}{\sqrt{2}}$ are good radiators and are called significant modes, whereas modes $MS<\dfrac{1}{\sqrt{2}}$ are non-significant modes^13^. Each mode at its resonant frequency contributes maximum towards the total current and radiations of the antenna and is called DM. The Resonant frequency of the mode is determined from the MS graph, a mode is resonant and called a DM at the MS peak. $\lambda _{n}$, $\alpha _{n}$, and MS values depend on the size and shape the of PEC structure and are independent of the source. The total current on the surface of the antenna structure at resonance is a combination of the currents of all modes that are coupled to the source^31^.

4 $$\begin{aligned} J_{total}= \sum _{n} a_{n} J_{n} \end{aligned}$$

5 $$\begin{aligned} a_{n}= \dfrac{V^i_{n}}{1+j\lambda _{n}} \end{aligned}$$

6Where $V^i_{n}$ is the modal excitation coefficient and $a_{n}$ is the modal weighting coefficient (MWC). $V^i_{n}$ defines the location of the source, whereas MWC depicts the amount of excitation of each mode in the presence of the applied source. MS identifies radiating capability while MWC depicts the excitation of a mode due to the source, together with these two parameters, the total contribution of each mode to the total antenna currents and radiations is measured. Nevertheless, a non-radiating mode with high MWC values has no contribution to the antenna radiations. Far fields associated with the induced modal currents according to CMA^29^ are given by:

7 $$\begin{aligned} E= \sum _{n} \dfrac{{V^i_{n}}{E_{n}}}{1+j\lambda _{n}} \end{aligned}$$

8 $$\begin{aligned} H= \sum _{n} \dfrac{{V^i_{n}}{E_{n}}}{1+j\lambda _{n}} \end{aligned}$$

Set of Eqs. (4)–(8) corroborates that: total antenna currents and radiations are directly proportional to two factors:

- Closeness of the modes to the resonant frequencies in the limit defined by CMA values $(\alpha _{n}/MS)$.
- Amount of the coupling of the mode to the source.

## Multiband antenna design using theory of characteristic modes

To highlight the performance benefit of the proposed technique, we have presented the antenna design using the two techniques. The following two approaches are outlined in this section

1. Excitation of only DMs for multiband antenna operation. In this approach, only one mode is excited in each band, so the currents and radiations of the antenna are shaped only by DM in each respective band.
2. Excitation and superposition of multiple modes at the same resonant frequency. In this approach, multiple modes are excited, therefore resultant antenna currents and radiations are the superposition of LOMs and DMs that are excited simultaneously at the same resonant frequency (superposition-based approach).

A flowchart explaining both methodologies is also provided.

**Figure 1:**
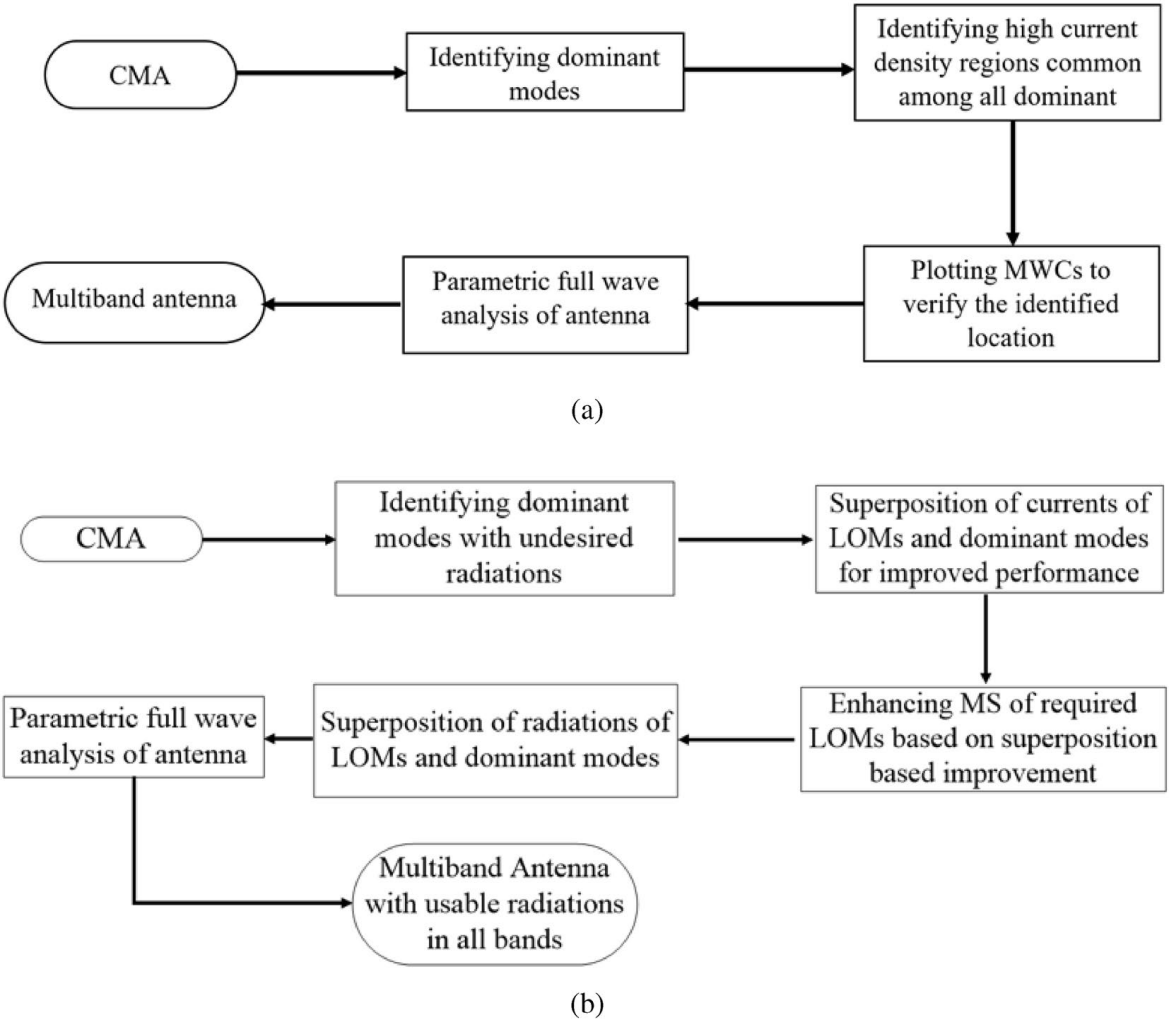
(**a**) Flowchart for multiband antenna design using DMs. (**b**) Flowchart for multiband antenna design using proposed technique of superposition of multiple modes at same resonance frequency.

### Flowchart multiband antenna design using dominant modes

A flowchart for the procedure to design a multiband antenna with the excitation of DMs is provided in Fig. 1a. The resonant frequency of DM is identified from the peak of MS curves. Surface currents of DMs are plotted at their resonant frequency, and modal currents are used to identify the feed point to excite DMs. A single feed point to excite all modes is identified by the high current density region that is common among all DMs. Modes excited by the source at the identified location are verified by the MWCs plot. In this approach, the resultant antenna has radiations and currents similar to the currents and radiations of DMs in their respective bands.

### Flowchart multiband antenna design using LOMs and DMs

A flowchart for the proposed technique which uses the superposition of DMs and LOMs is provided in Fig. 1b. In this approach, more than one mode is selectively excited at the same resonance, and the superposition of LOMs and HODM is performed to improve the radiations of antenna. Steps to locate feed point to excite LOMs with HODMs are: (1) identifying DMs with the undesired radiations, (2) identifying LOMs in the frequencies of DMs (3) analyzing surface currents and radiations after superposition of LOMs and HODMs (4) feeding antenna in the vicinity of the high-density current region that is common among all the required LOMs as well as DMs. The feed point is identified using the high-density currents of the dominant as well as the LOMs. Induced currents and radiations of multiband antenna designed using this approach are the superposition of all modes that are intended to be excited. In contrast to the antenna design using DMs, radiations of antenna using the proposed technique are shaped by the combined radiations of all modes that are intended to be excited at a single frequency in the respective bands.

## Characteristic mode analysis of partial Koch fractal

**Figure 2:**
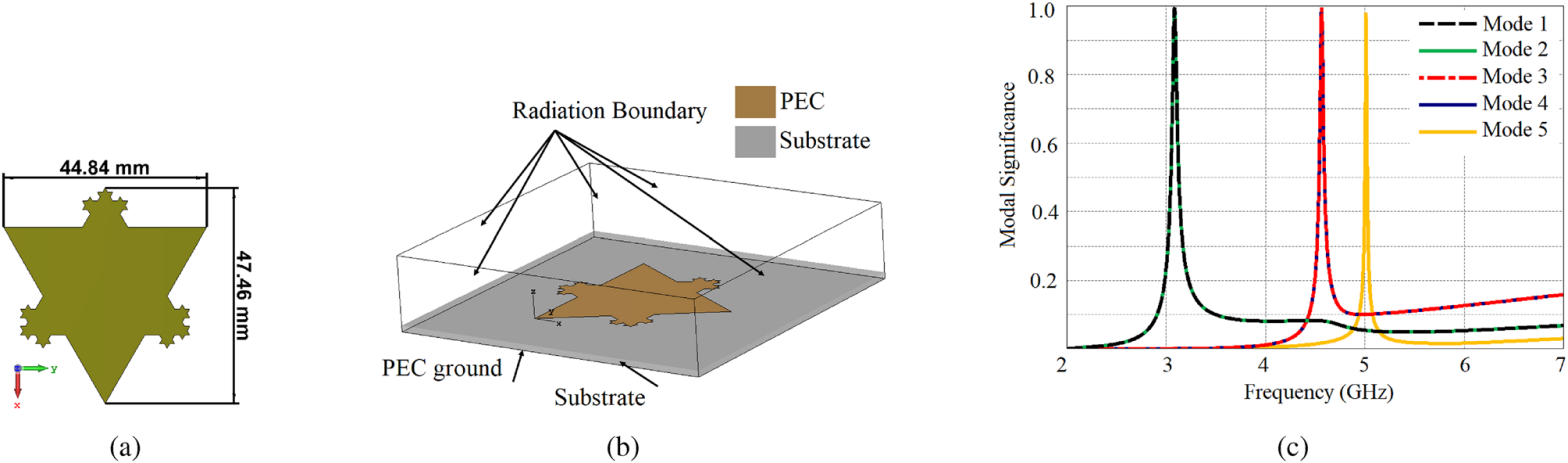
(**a**) Partial Koch Fractal, (**b**) Boundary conditions setup of antenna in CST, (**c**) MS variations with frequency.

To apply the two approaches delineated above, the CMA of antenna geometry^42^ provided in Fig. 2a, is carried out in the frequency range of 2–7 GHz, using CST Microwave Studio. As aforementioned, the Partial Koch fractal is chosen due to the inherent nulls in the radiations at high resonant frequencies^42, 43^, moreover it provides a large number of modes within the band. Simulations are performed with patch, lossless substrate (Rogers/Duroid 5870 $\epsilon _{r} = 2.33$) 1.5mm thickness, and infinite ground plane. To realize the radiation boundary, the problem domain is truncated by adding space in the five directions as shown in Fig. 2b, the perfect electric boundary is applied under the substrate layer to model an infinite PEC ground plane in the –z-direction. Five radiating modes are identified within the band in Fig. 2c, Due to the symmetry of the structure, two pairs of degenerate modes are resonant within the band. It can be seen in Fig. 2c, that one pair of fundamental degenerate modes is resonant at 3.15 GHz, the second pair of degenerate modes at 4.68 GHz, whereas the fifth mode is resonant at 5.10 GHz. It is observed that MS values of DMs right after their resonant frequencies are negligible depicting weak radiations of modes right after their resonant frequency. As a result, all DMs provide weak radiations after their resonant frequency, implying that antenna radiation patterns are shaped only by the radiations of DMs in their respective bands. It should also be noted that with the increase in frequency, the number of radiating modes is also increasing.

**Figure 3:**
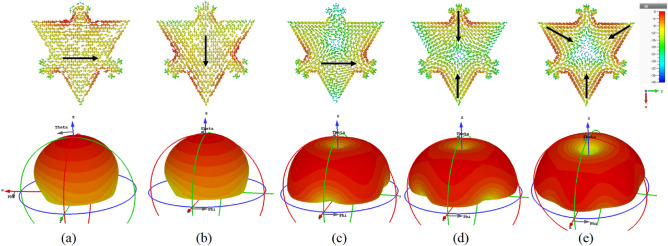
Modal currents and 3D radiations of DMs at their resonance (**a**) mode 1 at 3.15 GHz (**b**) mode 2 at 3.15 GHz (**c**) mode 3 at 4.68 GHz (**d**) mode 4 at 4.68 GHz (**e**) mode 5 at 5.10 GHz.

Surface currents and radiations of the DMs are provided in Fig. 3. Degenerate mode 1 &2 exhibit similar current symmetry with orthogonal relation, similarly, currents of degenerate mode 3 &4 are also orthogonally related. Mode 1 &3 has a horizontal surface current distribution, mode 2 &4 has vertical currents, whereas mode 5 has a combination of horizontal and vertical currents. Mode 1 &2 has maximum radiations in the broadside direction, a dip in the radiations of mode 3 &4, and a deep null in the radiations of mode 5 in the broadside direction of the antenna is observed in Fig. 3d. Null in the radiations is due to the currents flowing in the opposite direction, leading to cancelation in the center of the patch. Mode 1 &2 is desirable in multiband applications due to having maximum broadside radiations.

## Multiband antenna design using DMs

In this section, the antenna is designed using the procedure outlined in Fig. 1a. From the previous section, it is found that the CMA of the antenna provides two pairs of degenerate modes with the currents orthogonal to each other, thereby, providing two different alternatives in terms of the excitation of modes in the first and second bands. To design an antenna, modes are selected on the basis of similar current symmetries from each degenerate mode pair in the first and second bands. Using modes with similar symmetries provides more control over antenna currents and radiations. Therefore, two design examples of multiband antennas based on the selection of modes from the degenerate modes pairs are presented. Throughout the article, Antenna 1(a) will be used for antenna design using horizontal modes (mode 1 &3) with mode 5, and Antenna 2(a) for antenna design using vertical modes (mode 2 &4) with mode 5.

**Figure 4:**
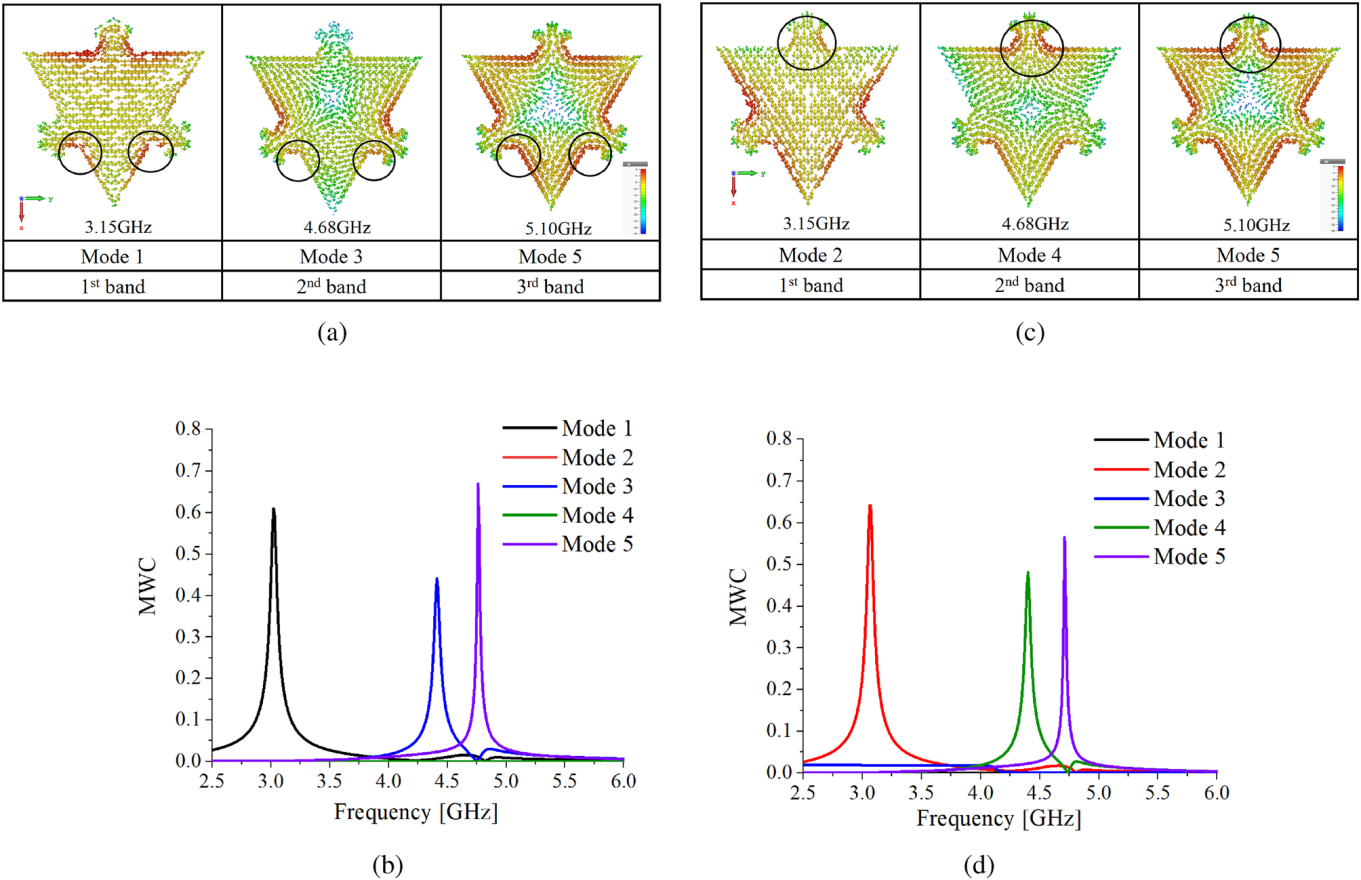
(**a**) Modes with horizontal current symmetries for multiband antenna design Antenna 1(a), (**b**) MWCs of Antenna 1(a), (**c**) Modes with vertical current symmetries for multiband antenna design Antenna 2(a), (**d**) MWCs of Antenna 2(a).

### Multiband antenna design using mode 1, 3, &5 Antenna 1(a)

In this section, a multiband antenna is designed with DMs having horizontal current symmetry i.e. modes 1, 3, & 5. Surface currents of mode 1, and 3 &5 at their resonant frequencies are plotted in Fig. 4a. To excite these modes, high current density regions common among modes is chosen as feed point as shown in the encircled region. To validate the feed point, CMA of the antenna is carried out after applying the source in the encircled region shown in Fig. 4a. Simulations are performed with the conditions provided in the previous section. From the MWCs values in Fig. 4b, we can see all DMs are simultaneously excited at their resonant frequencies, whereas the rest of the modes are completely suppressed. It should be noted that each mode after the MWC peak has negligible values indicating no impact on radiations after their resonance. Therefore, modal currents of Antenna 1(a) in three bands are given as

9 $$\begin{aligned} J_{3.15\;\text{GHz}}= a_{1}J_{1} \end{aligned}$$

10 $$\begin{aligned} J_{4.68\;\text{GHz}}= a_{3}J_{3} \end{aligned}$$

11 $$\begin{aligned} J_{5.10\;\text{GHz}}= a_{5}J_{5} \end{aligned}$$

Modal E-fields of Antenna 1(a) in three bands are provided by the set of equations

12 $$\begin{aligned} E_{3.15\;\text{GHz}}= a_{1}E_{1} \end{aligned}$$

13 $$\begin{aligned} E_{4.68\;\text{GHz}}= a_{3}E_{3} \end{aligned}$$

14 $$\begin{aligned} E_{5.10\;\text{GHz}}= a_{5}E_{5} \end{aligned}$$

Where $J_{1}$, $J_{3}$ and $J_{5}$ are the currents, and $E_{1}$, $E_{3}$, and $E_{5}$ are the fields of mode 1, 3, and 5 respectively. Whereas, $a_{1}$, $a_{3}$, and $a_{5}$ are the MWCs of modes 1, 3, and 5.

### Multiband antenna design using mode 2, 4, &5 Antenna 2(a)

To design a multiband antenna using vertical modes i.e. modes 2, 4, &5, the aforesaid procedure is repeated. CMA is carried out by applying a source in the encircled region as shown in Fig 4c. MWCs plotted in Fig. 4d depict the simultaneous excitation of modes 2, 4 & 5 at their resonant frequencies using a single source, whereas the rest of the modes are completely suppressed. Modal currents of Antenna 2(a) in three bands are provided

15 $$\begin{aligned} J_{3.15\;\text{GHz}}= a_{2}J_{2} \end{aligned}$$

16 $$\begin{aligned} J_{4.68\;\text{GHz}}= a_{4}J_{4} \end{aligned}$$

17 $$\begin{aligned} J_{5.10\;\text{GHz}}=a_{5}J_{5} \end{aligned}$$

Modal E-fields of Antenna 2(a) in three bands are provided by the set of equations

18 $$\begin{aligned} E_{3.15\;\text{GHz}}= a_{2}E_{2} \end{aligned}$$

19 $$\begin{aligned} E_{4.68\;\text{GHz}}= a_{4}E_{5} \end{aligned}$$

20 $$\begin{aligned} E_{5.10\;\text{GHz}}= a_{5}E_{5} \end{aligned}$$

Where $J_{2}$, $J_{4}$ and $J_{5}$ are the currents, and $E_{2}$, $E_{4}$, and $E_{5}$ are the fields of mode 2, 4 and 5 respectively. Whereas, $a_{2}$, $a_{4}$, and $a_{5}$ are the MWCs of mode 2, 4 and 5.

### Full-wave simulations of Antenna 1(a)

**Figure 5:**
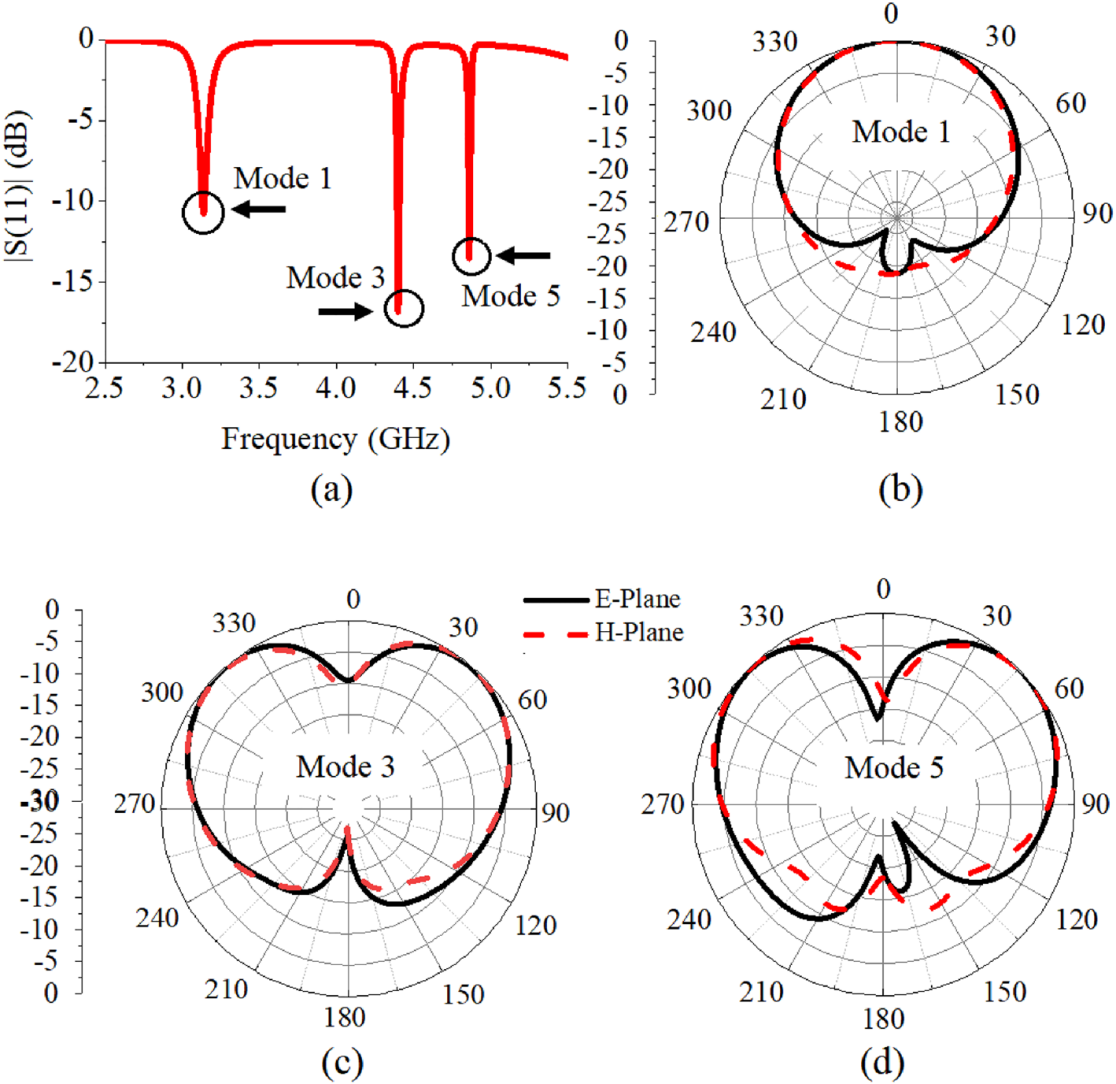
(**a**) Reflection coefficient of Antenna 1(a), 2D radiations of Antenna 1(a) in three bands (**b**) 3.10 GHz, (**c**) 4.45 GHz, (**d**) 4.85 GHz.

Analysis of the patch antenna is carried out in the frequency range of 2.5–5.5 GHz. Reflection coefficients and radiation patterns of the multiband Antenna 1(a) are shown in Fig. 5a–d. Simulations are performed with patch, substrate (Rogers/Duroid 5870 $\epsilon _{r}=2.33$) having dielectric loss tangent of 0.0012 and height of 1.5 mm, and ground plane of dimensions $80\; \text{mm} \times 80\; \text{mm}$. We can see that the antenna has maximum broadside radiations in the first band, while null in the second and third bands. It should be noted that radiation patterns in each band are fully characterized by the DMs in their respective bands. Nevertheless, in the second and third bands antenna has nulls in radiations which are very similar to the radiations of DMs in Fig. 3c,e. In order to resolve this issue, a technique based on excitation and superposition of multiple modes at the same resonance is proposed in the next section.

## Multiband antenna design using superposition of multiple modes at same resonant frequency

In this section, a design procedure based on the superposition of multiple modes at the same resonant frequency is provided, and as design example two antennas are presented. Currents of LOMs are combined with the HODMs to remove the current nulls, and superposition of subsequent radiations to provide enhancement in broadside radiations of the antenna in the second and third bands is achieved. Feed point location is identified using currents of both LOMs and HODMs. Antennas are designed by using the procedure outlined in Fig. 1b. Throughout the article, Antenna 1(b) will be used for the antenna excited by horizontal modes (mode 1 &3) with mode 5, whereas Antenna 2(b) for the antenna excited by the vertical modes (mode 2 &4) with mode 5, unless otherwise specified.

### Superposition of multiple modes for performance enhancement of Antenna 1(a) & Antenna 2(a)

Table 1 provides DMs at their resonant frequencies and LOMs within a band. It can be seen that there is no LOM in the frequency range of mode 1 &2, while two LOMs (mode 1 &2) are available in the resonant frequencies of the DMs 3 &4, while four LOMs i.e. mode 1,2, 3 &4 are available in the resonant frequencies of mode 5. To enhance antenna currents and radiations, one LOM from each pair of DMs is utilized in the resonant frequencies of the HODMs. The superposition of multiple modes is carried out to introduce current perturbations for the null removal in the broadside radiations of the antenna in the second and third bands. As mentioned previously, modes are selected based on their similar current symmetries.

**Table 1:** Dominant modes, and lower order modes within band.

DMs (n)	Resonant frequency (GHz)	LOMs (n)
1,2	3.9775	None
3,4	5.6225	1,2
5	5.9325	1,2,3,4

**Figure 6:**
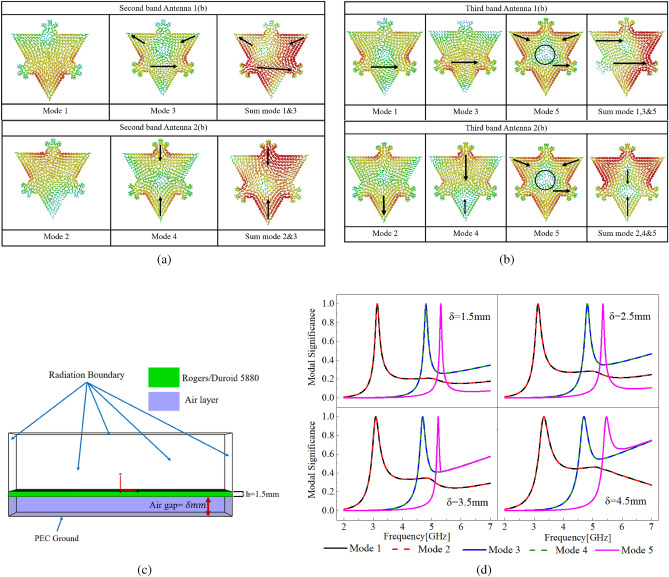
Modal currents at (**a**) 4.68 GHz, (**b**) 5.10 GHz, (**c**) Side view of the boundary conditions in CST, (**d**) MS of antenna at different air gap heights $\delta$.

To improve antenna performance in the second band, the superposition of LOMs and DMs as provided in Table 1 is carried out at 4.68 GHz. Total antenna currents provide strong current density on the overall patch structure and null in the center is minimized as is observed in the combined effect of mode 1 & mode 3 in Antenna 1(b) and mode 2 & mode 4 in Antenna 2(b) at 4.68 GHz shown in Fig. 6a, as compared with the currents of mode 3 & mode 4 at 4.68 GHz in Antenna 1(a) and Antenna 2(a) respectively shown in Fig. 3.

Similarly, to improve antenna performance in the third band, superposition of mode 1, 3 & 5 of Antenna 1(b) and mode 2, 4 & 5 of Antenna 2(b) is carried out at 5.10 GHz as shown in Fig. 6b. From the superposition of LOMs with the HODMs, we can see that the combined currents removed the cancellation of currents in the center of the patch, and as a result overall current density is increased. It is worth noting that as a result of the superposition of the currents in the second and third bands nulls in the currents are removed, nevertheless, this enhancement is not reflected in the radiations of the antenna due to negligibly small MS values of DMs right after their resonance. Small MS values of DMs depict weak radiating capability of the modes, therefore, to increase the contribution of modes towards radiations increase in the MS values is desired. In doing so, parametric CMA of antenna is performed with the air gap in next section to increase the radiating capability of LOMs in the frequencies of HODMs.

### Parametric analysis of Antenna 1(b) & Antenna 2(b)

To reveal the radiating mechanism of the antenna with excitation of multiple modes at the same resonant frequency, parametric analysis of the Partial Koch antenna at different air gap heights $(\delta )$ is carried out using CMA. The radiating capability of the non-significant LOMs is analyzed and enhanced in the frequencies of interest. The analysis is carried out using CST in the frequency range of 2–7 GHz, with the conditions provided in Fig. 6c.

**Figure 7:**
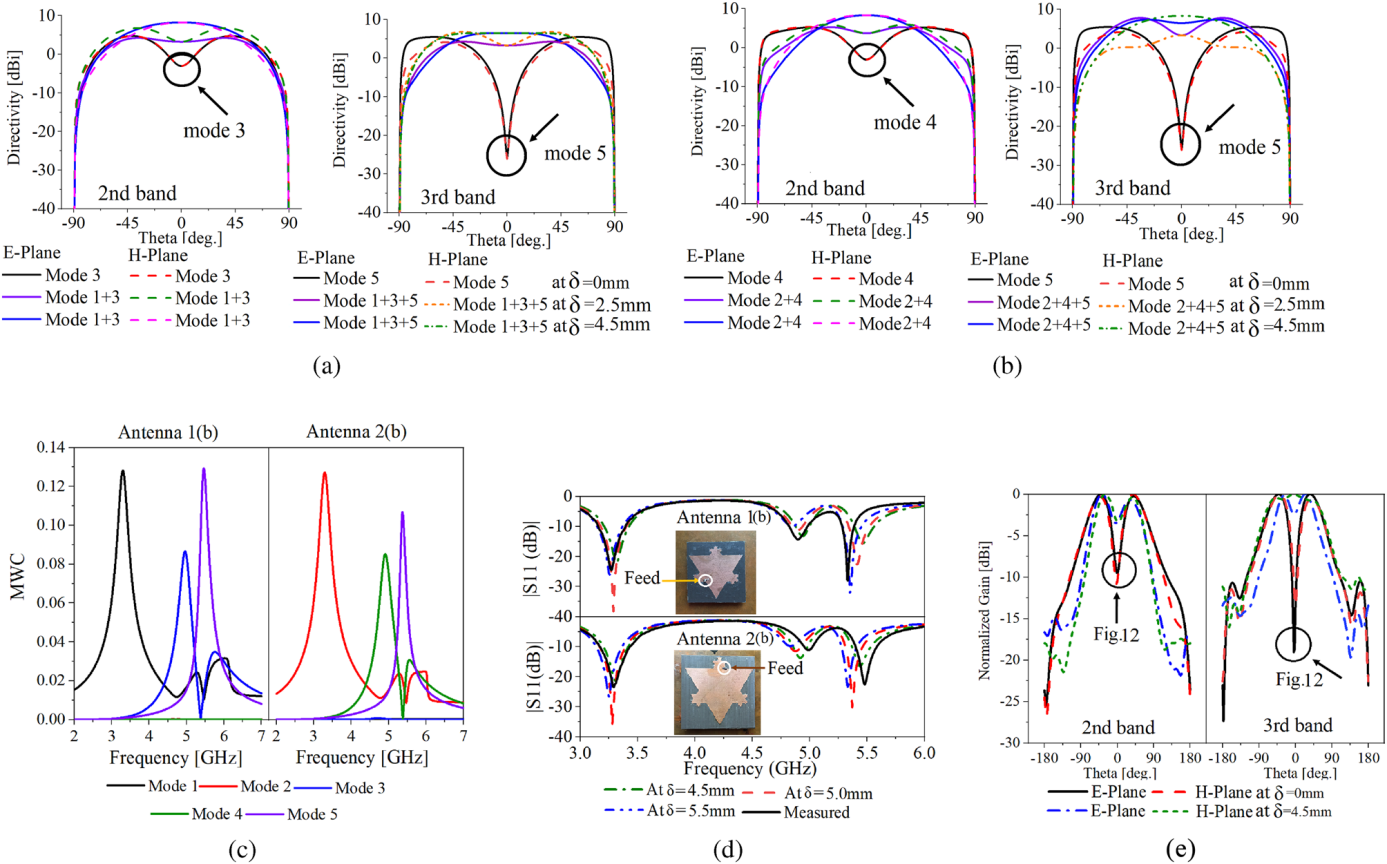
(**a**) 2D modal radiations in second and third band with and without superposition of multiple modes of Antenna 1(b), (**b**) 2D modal radiations in second and third band with and without superposition of multiple modes of Antenna 1(b), (**c**) MWCs at $\delta =4.5\;\text{mm}$, (**d**) Reflection coefficient, and fabricated prototypes of Antenna 1(b) Antenna 2(b), (e) Simulated normalized gain comparison of Antenna 1(a) and Antenna 1(b).

Progression of the radiating behavior of LOMs with the increase in air gap heights $\delta$ between the ground plane and the substrate is provided in Fig. 6d. It is shown that, with the increase in $\delta$, MS values of DMs also increase beyond their resonant frequencies. Analysis of antenna at different air gap heights $\delta$ also depicts the slight increase in the bandwidths of modes. An increase in the MS values after the peak due to an increase in the overall height of the patch shows that the radiating capability of DMs after their resonance gradually increased with the subsequent rise in the height of the patch. We can see that MS values of mode 1 &2 (LOMs) at the resonant frequency of mode 3 &4 increased from 0.08 at $\delta =0\;\text{mm}$ to 0.57 at $\delta =4.5\;\text{mm}$. MS values of mode 1 &2 (LOMs) at the resonant frequency of mode 5 increased from 0.05 at $\delta =0\;\text{mm}$ to 0.45 at $\delta =4.5\;\text{mm}$, whereas mode 3 &4 (LOMs) increased from 0.1 at $\delta =0\;\text{mm}$ to 0.56 at $\delta =4.5\;\text{mm}$. Slight variation in the resonant frequencies of the modes is also observed with the variations in the height of the patch since the increase in the air gap height reduces effective permittivity. Due to the incremental variation in the MS values with the increase in the height of the patch, a direct relationship between the modal contribution of LOMs is established with the antenna thickness.

2D rectangular modal directivity comparison graph of Antenna 1(b) & Antenna 2(b) is provided in Fig. 7a,b respectively. Modal directivity comparison depicts significant improvement in the antenna radiations after the superposition of LOMs with the HODMs in the second and third bands. Deep null in the antenna radiations in the second and third bands is removed after the superposition of LOMs in the resonant frequencies of HODMs (mode 3 &4, mode 5). Directivity enhancement in the broadside direction of the antenna after superposition is provided in Table 2, an overall enhancement of 13dBi in the broadside radiations of the antenna in the second band, while 36dBi in the third band is achieved using superposition of modes.

**Table 2:** Modal directivity enhancement in the broadside direction of Antenna 1(b) & Antenna (2b).

Improvement in the second band		Improvement in the third band	
$\varphi =0^{0}$	$\varphi =90^{0}$	$\varphi =0^{0}$	$\varphi =90^{0}$
13 dBi	13 dBi	32 dBi	32 dBi

To excite the required modes simultaneously at the same resonant frequency, a high current density region that is common among all DMs and LOMs is chosen as the feed point, and CMA of the antenna with the source is carried out in the frequency range of 2–7 GHz. Simulations are performed with infinite ground plane conditions, the substrate used is lossless Rogers Duroid 5870 with permittivity of 2.33, the thickness of the substrate used is 1.5 mm, and an air gap of 4.5 mm is used between the substrate and ground plane, and coaxial probe is used. From the MWCs in Fig. 7c, we can see that LOMs and HODMs are simultaneously excited using a single source. In Antenna 1(b) mode 1 is excited at the first resonance, mode 1 &3 is excited at the second resonance, whereas modes 1,3 &5 are excited at the third resonance of the antenna. Similarly, in Antenna 2(b) mode 2 is excited at the first resonance, mode 2 &4 is excited at the second resonance, whereas modes 2,4 &5 are excited at the third resonance of the antenna. Modal currents of Antenna 1(b) in three bands, are represented by the following set of equations

21 $$\begin{aligned} J_{3.15\;\text{GHz}}= a_{1(3.15\;\text{GHz})}J_{1(3.15\;\text{GHz})} \end{aligned}$$

22 $$\begin{aligned} J_{4.68\;\text{GHz}}= a_{1(4.68\;\text{GHz})}J_{1(4.68\;\text{GHz})}+a_{3(4.68\;\text{GHz})}J_{3(4.68\;\text{GHz})} \end{aligned}$$

23 $$\begin{aligned} J_{5.10\;\text{GHz}}= a_{1(5.10\;\text{GHz})}J_{1(5.10\;\text{GHz})}+a_{3(5.10\;\text{GHz})}J_{3(5.10\;\text{GHz})}+a_{5(5.10\;\text{GHz})}J_{5(5.10\;\text{GHz})} \end{aligned}$$

Modal E-fields of Antenna 1(b) in three bands are given as

24 $$\begin{aligned} E_{3.15\;\text{GHz}}= a_{1(3.15\;\text{GHz})}E_{1(3.15\;\text{GHz})} \end{aligned}$$

25 $$\begin{aligned} E_{4.68\;\text{GHz}}= a_{1(4.68\;\text{GHz})}E_{1(4.68\;\text{GHz})}+a_{3(4.68\;\text{GHz})}E_{3(4.68\;\text{GHz})} \end{aligned}$$

26 $$\begin{aligned} E_{5.10\;\text{GHz}}= a_{1(5.10\;\text{GHz})}E_{1(5.10\;\text{GHz})}+a_{3(5.10\;\text{GHz})}E_{3(5.10\;\text{GHz})}+a_{5(5.10\;\text{GHz})}E_{5(5.10\;\text{GHz})} \end{aligned}$$

Where $J_{1}$, $J_{3}$ and $J_{5}$ are the currents, and $E_{1}$, $E_{3}$, and $E_{5}$ are the fields of mode 1, 3, and 5 at the specified frequencies respectively. Whereas, $a_{1}$, $a_{3}$, and $a_{5}$ are the MWCs of modes 1, 3, and 5 at the frequencies specified respectively. Modal currents of Antenna 2(b) in three bands are as under

27 $$\begin{aligned} J_{3.15\;\text{GHz}}= a_{2(3.15\;\text{GHz})}J_{2(3.15\;\text{GHz})} \end{aligned}$$

28 $$\begin{aligned} J_{4.68\;\text{GHz}}= a_{2(4.68\;\text{GHz})}J_{2(4.68\;\text{GHz})}+a_{2(4.68\;\text{GHz})}J_{2(4.68\;\text{GHz})} \end{aligned}$$

29 $$\begin{aligned} J_{5.10\;\text{GHz}}= a_{2(5.10\;\text{GHz})}J_{2(5.10\;\text{GHz})}+a_{4(5.10\;\text{GHz})}J_{4(5.10\;\text{GHz})}+a_{5(5.10\;\text{GHz})}J_{5(5.10\;\text{GHz})} \end{aligned}$$

Modal E-fields of the Antenna 2(b) in three bands are provided

30 $$\begin{aligned} E_{3.15\;\text{GHz}}= a_{2(3.15\;\text{GHz})}E_{2(3.15\;\text{GHz})} \end{aligned}$$

31 $$\begin{aligned} E_{4.68\;\text{GHz}}= a_{2(4.68\;\text{GHz})}E_{2(4.68\;\text{GHz})}+a_{4(4.68\;\text{GHz})}E_{4(4.68\;\text{GHz})} \end{aligned}$$

32 $$\begin{aligned} E_{5.10\;\text{GHz}}= a_{2(5.10\;\text{GHz})}E_{2(5.10\;\text{GHz})}+a_{4(5.10\;\text{GHz})}E_{4(5.10\;\text{GHz})}+a_{5(5.10\;\text{GHz})}E_{5(5.10\;\text{GHz})} \end{aligned}$$

Where $J_{2}$, $J_{4}$ and $J_{5}$ are the currents, and $E_{2}$, $E_{4}$, and $E_{5}$ are the fields of mode 2, 4 and 5 at the specified frequencies respectively. Whereas, $a_{2}$, $a_{4}$, and $a_{5}$ are the MWCs of modes 2, 4, and 5 at the above-mentioned frequencies respectively.

### Parametric analysis of Antenna 1(b) & Antenna 2(b)

Parametric analysis of Antenna 1(b) and Antenna 2(b) is performed with the same conditions as provided before. The analysis includes optimization of reflection coefficients of both antennas in three bands. Simulated reflection coefficients of Antenna 1(b) and Antenna 2(b) at different air gap heights $\delta$ are shown in Fig. 7d. In both antennas coaxial-probe is connected in the vicinity of regions as verified by plotting the MWCs in Fig. 7c. It can be seen that all modes are simultaneously excited using a single source. A slight shift of resonant frequencies is observed, which is due to the inclusion of the lossy substrate, and change in the location of the feed point for optimization and better matching of the antenna in three bands.

Fabricated prototypes of Antenna 1(b) and Antenna 2(b), and measured reflection coefficients are provided in Fig. 7d. A slight variation in the measured and simulated reflection coefficient is due to the material losses and fabrication error. The measured bandwidth, directivity, and gain of Antenna 1(b) and Antenna 2(b) are provided in Table 3.

**Table 3:** Measured bandwidth, directivity, and gain.

Antenna	Resonant frequency (GHz)	Directivity (dBi)	Gain (dBi)
1(b)	3.15–3.34 (0.19)	9.82	**8.99**
	4.80–4.98 (0.18)	7.61	6.97
	5.26–5.39 (0.13)	6.98	6.40
2(b)	3.15–3.43 (0.28)	9.74	8.90
	4.95–5.05 (0.10)	8.16	7.45
	5.39–5.58 (0.19)	8.27	7.59

**Table 4:** Gain enhancement in the broadside direction of Antenna 1(b) as compared to Antenna 1(a).

Improvement in the second band		Improvement in the third band	
$\varphi =0^{0}$	$\varphi =90^{0}$	$\varphi =0^{0}$	$\varphi =90^{0}$
10 dBi	10 dBi	16 dBi	20 dBi

**Figure 8:**
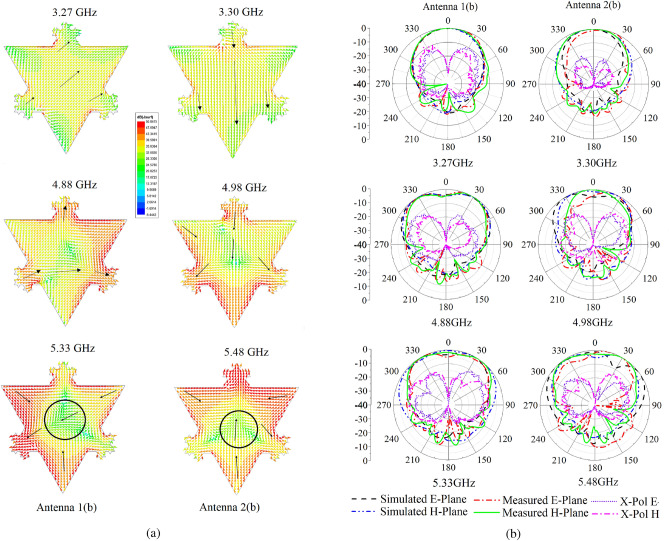
(**a**) Surface currents of Antenna 1(b) & Antenna 2(b)), (**b**) Normalized simulated and measured radiation patterns.

A comparison of simulated 2D normalized gain patterns of Antenna 1(a) (Fig. 5) and Antenna 1(b) in the second and third bands is provided in Fig. 7e depicts a significant improvement in the radiation patterns of Antenna 1(b). Gain enhancement is achieved without introducing slots. Table 4 provides the gain enhancement in the broadside direction of the antenna in the second and third band. Simulated currents in three bands are provided in Fig. 8a. It can be seen that the null in the middle of total antenna currents is removed. Total antenna currents in Fig. 8a are in good agreement with the modal currents of Fig. 3a,b, and combined currents in Fig. 6a and b. Small variations in the currents are due to the inclusion of lossy substrate and source. Measured radiation patterns are in good agreement with the simulated patterns as shown in Fig. 8b, slight variations in the radiations are due to fabrication errors. Low cross-polarization is achieved in all bands, and nulls in the second and third bands are removed.

## Comparison table

To compare the proposed technique with the other methods, a performance comparison is provided in Table 5. The performance of both antennas is compared with other multiband antennas. Sizes are calculated at the lowest frequency of the band, while gains are provided at the lowest frequencies in the first column. From the data, it is concluded that better gain performance of both antennas is achieved in all bands while maintaining the lower profile of $0.063 \lambda _{L}$.

**Table 5:** Performance comparison of multiband patch antennas.

Ref [n]	Antenna type	Frequency (GHz)	Bandwidth (%)	Gain (dBi)	Null in Broadside Radiations	Thickness ($\lambda _{L}$)	Size ($\lambda _{L}*\lambda _{L}$)
^3^	SRR fractal patch	5.0/6.8/7.5/8.5	4/3.24/2.93/3.29	Negative/negative/12.5/13.3	Yes	0.0267	0.75 * 0.75
^4^	Sierpinski fractal antenna	0.9/1.8/2.4	8.44/6.67/8.20	0.34/4.11/2.75	No	0.0024	0.210 * 0.161
^5^	H fractal antenna	0.36/1.32/5.5	N.A	1.91/3.72/7.52 (peak directivities)	Yes, 2nd and 3rd band	0.0019	0.144 * 0.123
^11^	Slotted patch	5.0/5.8/6.55	7.6/5.5/8.9	8	No	0.08167	0.359 * 0.249
^12^	Slotted patch with vias	2.98/4.73/5.7	Narrowband/% N.A	2.59/3.58/2.29	No	0.0195	0.129 * 0.129
^13^	Slotted patch with varactor diodes	2.33/3.21/4.99	23.55/22.08/16.03	2.72/2.76/2.87	No	0.0135	0.10 * 0.17
^37^	Fractal patch	1.25/2.1	Narrowband/% N.A	6	No	0.121	0.208 * 0.208
^39^	Slotted metasurface	26.5/38.9	20.7/11.3	7.2/10.9	No	0.1807	0.832 * 0.832
Current work Antenna 1(b)	Partial fractal patch	3.27/4.88/5.33	5.855/3.681/2.441	8.99/6.97/6.40	No	0.063	0.470*0.498
Current work Antenna 2(b)	Partial fractal patch	3.30/4.98/5.48	8.511/2.00/3.464	8.90/7.45/7.59	No	0.063	0.470 * 0.498

($\lambda _{L}$) is the wavelength at lowest frequency.

## Conclusion

A procedure is proposed to design antenna using MC of LOMs at the resonant frequencies of HODMs for multiband antenna operation with improved radiation performance and control. The radiating capability of modes having poor radiations is enhanced beyond their resonance to shape the broadside radiations of antenna in multiple bands. The carefully chosen feed point is identified to excite desired modes with the required contributions. As a proof of concept, this technique is utilized to design a multiband patch antenna with stable and enhanced radiations. The concept is verified by achieving stable and enhanced radiations in all bands, also null in the radiations of the second and third bands is successfully removed. Close agreement is observed between simulated and measured results. The proposed procedure proves that multiband behavior with improved radiations can be achieved by properly using the MC of LOMs in the resonant frequencies of HODMs. Required results are achieved without any modifications in the structure of the patch or designing complex feed structure.

## Data Availability

All data has been added in this study.
